# Women in Rheumatology in the Arab League of Associations for Rheumatology Countries: A Rising Workforce

**DOI:** 10.3389/fmed.2022.880285

**Published:** 2022-06-09

**Authors:** Nelly Ziade, Ihsane Hmamouchi, Lina El Kibbi

**Affiliations:** ^1^Rheumatology Department, Hôtel-Dieu de France Hospital, Saint Joseph’s University, Beirut, Lebanon; ^2^Rheumatology Unit, Temara Hospital, Laboratory of Biostatistics, Clinical Research and Epidemiology (LBRCE), Faculty of Medicine and Pharmacy, Mohammed V University, Rabat, Morocco; ^3^Rheumatology Unit, Internal Medicine Department, Specialized Medical Center, Riyadh, Saudi Arabia

**Keywords:** rheumatology, workforce, leadership, female, women empowerment, social media

## Abstract

**Background:**

An increase in women physicians in the medical workforce is witnessed in recent decades, paralleled by an increase in leadership positions and, to a lesser extent, in academic advancement.

**Objectives:**

This study aims to evaluate the women rheumatologists (WR) workforce and to identify the challenges faced by WR in the Arab League of Associations for Rheumatology (ArLAR) countries.

**Methods:**

We collected data from members of special interest groups from 16 ArLAR countries about the number of rheumatologists in the national societies and boards – including WR –, and the estimated percentage of WR involved in academia. Also, a sample of WR was identified based on their established leadership positions and invited to a structured interview addressing their career path and the gender-related challenges.

**Results:**

The proportion of WR varied widely across the ArLAR countries, with a mean of 56%. Moreover, WR constituted 47% of the society’s board members and roughly 49% of WR were involved in academia. However, only 37% of the current society presidents were females. Structured interviews indicated that WR place a high value on patient care and teaching, followed by research and publications. The primary reported gender-related challenge was balancing work with family demands. Moreover, some WR reported gender-related discrimination incurred by colleagues, patients, and administrations.

**Conclusion:**

WR constituted more than half of the current rheumatology workforce in the ArLAR countries, with a lower – but steadily growing – proportion of WR in leadership positions. As they embrace their growing role in the workforce, WR must benefit from all the provided tools, from learning from the experience of current women leaders in the field to using the latest technology such as social media platforms to empower them to reach gender equity.

## Introduction

Gender disparities in medicine have always been a subject of debate, as women have faced several challenges in achieving gender equity, especially in reaching and maintaining leadership positions and academic advancement (1). However, an increase in women physicians in the medical workforce is witnessed in recent decades (2–4), and this rise in the workforce is mirrored by an increasing proportion of women becoming program directors and division heads in different branches of medicine (5). However, in academia, women are still less likely to be promoted to the rank of associate or full professor and have fewer publications with first and senior authorship (6). Moreover, women are under-represented as first and senior authors for publications reporting industry-sponsored studies and randomized controlled trials (7), whereas gender parity is more balanced in the first authorship for investigator-led research publications. One of the reasons behind this discrepancy could be that women engage in clinician-educator tracks rather than research tracks (1), and that they take a less important part in industry-collaborative research than men.

In the Arab countries, the number of female physicians has also been steadily increasing with time, which is paralleled by an increase in leadership positions (8, 9) in different medical specialties. However, according to our knowledge, there are no specific studies about the female rheumatology workforce in the Arab countries of the Middle East and North Africa (MENA) region. The Arab League of Associations for Rheumatology (ArLAR) includes 16 countries with 384 million inhabitants. These countries extend over 13 million km^2^, 2 continents (Asia and Africa), and 4 time zones (UTC+0 to UTC+4) (10, 11) and share the same language and some cultural similarities. Notably, these countries are connected through an organized network that facilitates the conduction of collaborative research.

This study aims to evaluate the women rheumatologists (WR) workforce and to identify the challenges faced by WR in the ArLAR countries.

## Materials and Methods

### Women Rheumatologists Workforce

For the purpose of this study, the workforce was defined as the WR practicing in ArLAR countries. Rheumatology fellows, nurses, and assistants were not included in the current analysis. Data were collected in 16 Arab countries that are part of the ArLAR in November and December 2021.

The data regarding the number of rheumatologists registered in the national societies and the number of rheumatologists in the national society boards – including the number of WR specifically – and the estimated percentage of WR involved in academia were requested by e-mail from society members working in special interest groups within the ArLAR. In addition, the gender of the past and current president of the national society was recorded. Finally, the size of the population by country was retrieved from the 2020 World Bank source (12). The data were presented descriptively using numbers and percentages.

### Current Challenges for Women in Rheumatology

Women rheumatologists from 17 Arab countries (16 ArLAR countries and Bahrain) were identified based on their current or past known leadership positions (past or current society presidents, society board members, and WR with high academic positions such as dean of medicine). The selection of the rheumatologists was based on a convenience sampling technique, closely related to their leadership positions, associated to a quota sampling technique, where at least one woman rheumatologist was invited from each Arab country. Each WR was interviewed by one of the authors using a live interview on the Zoom platform. In case of the impossibility of doing a live interview, responding by e-mail was proposed. The interview was based on a structured questionnaire including 12 questions compiled from previous interviews conducted by the authors (Supplementary Data). The questionnaire comprised two parts: the first one corresponding to the rheumatology career in general and the second part to the specific challenges faced due to the female gender. The questions were formulated in English, and the interview was conducted in mixed language (English and Arabic). Data were collected over 2 months (November and December 2021).

All the answers were transcribed in a Microsoft Excel document and classified by question. There was no specific software used in the qualitative analysis. Thereafter, a qualitative analysis of the responses to each question was done separately. The answers were analyzed by the authors and grouped into homogeneous themes or domains. The number of times a theme recurred was also recorded. Data was published anonymously.

## Results

### Women Rheumatologists Workforce

Globally, the Arab countries have 3,454 registered rheumatologists for a total of 382 million inhabitants, indicating a proportion of 0.90 rheumatologists per 100,000 inhabitants (or one rheumatologist for 110,596 inhabitants). However, this proportion varied widely among countries and ranges from 0.23 in Libya to 1.8 rheumatologists per 100,000 inhabitants in Tunisia (Table 1).

**TABLE 1:** Rheumatology workforce in the ArLAR countries.

Country	Country population (in millions)	Number of rheumatologists in the national society	Number of WR in the society (% from all rheumatologists)	Number of rheumatologists in the society board	Number of WR in the society board (% from all board)	Percentage of WR active in academia	Sex of the past society president	Sex of the current society president
Algeria^#^	43.8	614	260 (42.4%)	23	14 (60.9%)	50%	Male	Female
Egypt	102.3	1,388	944 (68.0%)	14	3 (21.43%)	89%	Male	Male
Iraq	40.2	263	88 (33.5%)	7	1 (14.3%)	30%	Male	Male
Jordan	10.2	34	8 (23.5%)	5	1 (20.0%)	15%	Male	Male
Kuwait	4.3	35	18 (51.4%)	4	3 (75.0%)	50%	Female	Female
Lebanon	6.8	51	18 (35.3%)	7	3 (42.9%)	39%	Male	Male
Libya	6.9	16	11 (68.8%)	5	3 (60.0%)	40%	Male	Female
Morocco	36.9	370	239 (64.6%)	14	12 (85.7%)	62%	Male	Male
Oman	5.1	15	6 (40.0%)	7	2 (28.6%)	50%	Female	Male
Palestine	4.8	14	1 (7.1%)	4	0 (0%)	100%	Male	Male
Qatar	2.9	30	10 (33.3%)	10	3 (30.0%)	50%	NA*	Female
Saudi Arabia	34.8	246	96 (39.0%)	9	2 (22.2%)	NA	Male	Female
Sudan	43.8	44	27 (61.4%)	4	4 (100%)	33%	Female	Male
Syria	17.5	64	40 (62.5%)	7	3 (42.7%)	17%	Female	Female
Tunisia	11.8	220	156 (70.9%)	5	4 (80.0%)	66%	Male	Male
UAE*	9.9	50 (approximate)	NA	13	7 (53.8%)	NA	Male	Male
Total	382	3,454	1,922 (56.5%)	138	65 (47.1%)	49.4%	4 female/15 26.7%	6 female/16 37.5%

*NA, not available; WR, women rheumatologists.*

*^#^Data pooled from three societies.*

*Data pooled from two societies.

The proportion of women within the national rheumatology societies was 56.46% (1,922/3,404), ranging from 7.14% in Palestine to 70.91% in Tunisia, with a median of 42.35%.

Within the national society board committees, women constituted 47.1% of all board members (65/138), with a wide range going from 0% in Palestine to 100% in Sudan and a median value of 42.86%.

The percentage of WR involved in academia was estimated to be 49.33%, ranging from 15% in Jordan to 100% in Palestine (based on only one woman rheumatologist).

As for the leadership position within the national society, the past president was a female in 26.7% of the societies (4 presidents in 15 countries), whereas the current president is a female in 37.5% of the societies (6 presidents in 16 countries) (Table 1).

### Current Challenges for Women in Rheumatology

**FIGURE 1:**
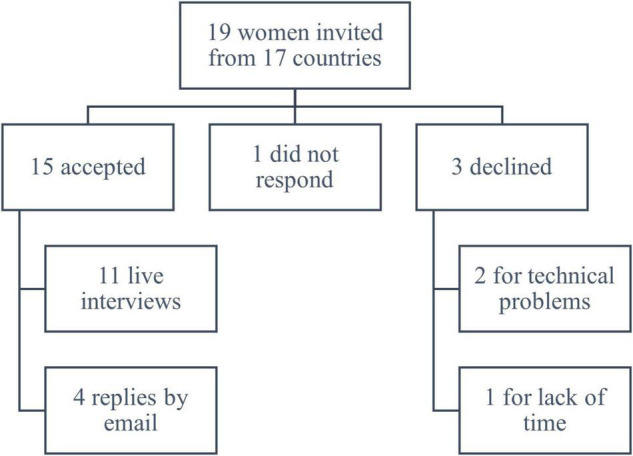
Flowchart of interview participants from the 14 ArLAR countries.

The authors invited 19 WR to participate in the qualitative structured interview; 15 rheumatologists from 14 Arab countries accepted (78.95%), 1 did not respond, and 3 declined for technical problems or lack of time (Figure 1). The interview was performed live over Zoom with 11 WR and over email with 4 WR. The live interviews’ duration ranged ted from 17 to 21 min, with an average duration of 19 min. The participants’ age ranged from 45 to 70 years. Two of the interviewees were previous dean of medicine, five were current Societies’ presidents, five were head of departments, and were three Societies’ president-elect. All of the interviewees were involved in academic activities, in addition to their clinical work, and they were from the following countries: Algeria, Bahrain, Egypt, Iraq, Jordan, Kuwait, Lebanon, Libya, Morocco, Qatar, Saudi Arabia, Sudan, Syria, and Tunisia.

Regarding their career in general, the first reason for choosing rheumatology was the intellectual interest in the specialty *per se*, considering it as challenging, analytical, and multisystemic. Some WR chose rheumatology because of an unmet need for this specialty in their respective countries, and a few chose it because of the convenient lifestyle (Table 2). In 13 WR, the influence of a mentor who had an impact on their career choice was reported. Most of the time, the mentor was a male, and his origin was balanced between local and international.

**TABLE 2:** Summary of the interviews with women rheumatologists from the ArLAR countries regarding the rheumatologist’s career journey.

Question	Summary of responses (number of response recurrence)
How did you become interested in rheumatology?	Intellectual interest in the specialty: challenging, multisystemic, analytical (8) Unmet need in the country (4) Convenient lifestyle (3) Personal or familial musculoskeletal experience (2) Specific patients’ cases (2) Research opportunities, choice by default
Did you have any mentors?	International mentor (5), local mentor (4), both local and international mentors (4) No mentor (2)
What are the top 3 elements that shaped your career?	Dedication for patients (5) Parent/partner support (4) International training and networking (4) Discipline and commitment (3), continuous medical education (3) Training in research (3) Ambition (3) and love for the challenge (3) Love of transmitting knowledge/teaching (3) Personal experience as a patient (1), ethics and religious beliefs (1), having a good mentor (1)
What 5 skills helped you to be a role model and inspiring doctor?	Professional and committed (7) Good communicator and good listener (6) Good team player (5) Have good work ethics and respect for patients (5), empathic (5) Continuously updating knowledge (5) Enthusiastic and motivated for work (4), passion for teaching and sharing knowledge (4) Patient (3), positive and confident (3), humble, friendly, and kind (3) Strategic thinker (2) Ambitious (1), strong (1), leader (1)
What has been your main achievement so far?	Teaching of young rheumatologists and being a role model for students and colleagues (9) Having success within the national rheumatology society (8) Being a member of an international organization/network (7) Promoting research and publications (6) Establishing a rheumatology unit/managerial position (6) Assuming an academic leadership position (4) Raising a family (3) Promoting patient education (2)
What is your current professional focus?	Improving research (10), improving rheumatology training (5) Assuming an academic leadership position (4) Promoting and being an advocate for rheumatology (3) Leading national and regional organization (ArLAR) (3) Managing a rheumatology unit (3), promoting patient education (3) Improving personal skills (such as musculoskeletal ultrasound) (1) Narrowing the gender gap in rheumatology (1)
What is next for you? Is there a dream job as a rheumatologist?	Four persons state that they have reached their highest potential Boost research and publications (5) Promote rheumatology in the country (3) and the region (2) Develop a rheumatology excellence center (2), establish a private career (2) Leading the national society (1), teach the new generation (1) Establish a women-based task force to support women research and training (1)

Very diverse elements shaped the WR’s careers, topped by the dedication to patients as a first component. Also, many WR reported the importance of their parent or partner’s support, the role of international training and networking, and the added value of training in research and epidemiology.

Among the most cited skills that helped the WR be a role model and inspiring doctor were professionalism and commitment. In addition, communication was considered an important skill, as well as being a good team player, having good work ethics, and being empathic. Moreover, continuous update of knowledge was considered an essential personal skill.

When asked about their main achievement so far, most WR cited their teaching role, transmitting knowledge to young rheumatologists and being a role model for them. Also, many WR considered their active part in national and international societies as a significant achievement. Moreover, promoting research and publications was considered the main achievement in many.

The current professional focus of most WR was improving research, followed by improving rheumatology training and assuming an academic position.

As for the challenges that the interviewees faced because of being women (Table 3), it is interesting to note that four women reported no gender-related obstacles. For the rest of WR, the primary obstacle was balancing work with family demands, leading to a need to work more to prove oneself and to loss of career opportunities in some cases. The WR also faced reluctance from male colleagues to have a woman leading the team and from male patients to gain their confidence and be at ease during a clinical exam. However, some women reported that female patients were more likely to consult a female physician, which has been an advantage since most rheumatic diseases are more prevalent in females. Also, WR reported under-appreciation and delay in career advancement from the administration because of their gender.

**TABLE 3:** Summary of the interviews with women rheumatologists from the ArLAR countries regarding the challenges faced as a woman and how to overcome them.

Question	Summary of responses
Did you face any obstacles because you are a woman? If yes, name the top 3.	Four out of 15 women said that there were no obstacles during their careers. Balancing work with family demands (6) Reluctance from male colleagues to have a woman leading the group (3) Getting the confidence of male patients and examining them (2) Under-appreciation, and delay in career advancement from administration (2) Criticism over being a woman active on social media
How did you manage your work-life balance?	Time management and early planning (11) Delegating to teams at work and to the family at home (5) Support from husband (5) from family (5) Need to do compromise and set priorities (5) Have a dedicated time for family as a priority (4) Have personal time (4), for example, sports activities, reading, exercising, traveling, and hobbies Compartmentalization of personal and professional life
Do you have any tips for the new rheumatologists about that?	Be confident (2), set goals (2) Be strong, persistent, motivated Accept failure, update knowledge
How do you see the future of women rheumatologists in our region?	Very qualified current workforce (5) and promising future for WR (9) Collaboration among countries to make a higher impact No concern about women but concern about rheumatology as a threatened discipline (2) Although a majority, 30% of WR do not work because of family or because they cannot afford to have a private clinic Universities do not believe in women taking leadership positions

Nevertheless, WR managed to reach a good work-life balance using time management, early planning, delegating, and having support from the family, although they reported that many compromises had to be made, and priorities had to be set. They provided some motivational advice for the new generation: “Have faith,” “Set your goals, be persistent, and you will fulfill your dreams.” Most of them are satisfied with the current status of WR in the ArLAR countries and are very optimistic about their future. However, since a higher proportion of rheumatologists are women today, and since WR tend not to work or work only as part-time (30% as per one’s estimation), and due to many other non-gender-related challenges, they are concerned about the future of rheumatology as a discipline. Therefore, they propose that women and men rheumatologists join forces to advocate strongly for their specialty.

## Discussion

In the ArLAR countries, the proportion of WR (56% in the current study) was higher than the ones reported in other countries: 52% in Canada in 2021 (13), 50% in Australia and New Zealand in 2019 (14), 49% in Latin America in 2020 [49.2% (15)], 47% in the United Kingdom in 2018 (16), and 41% in the United States in 2015 (2). Nevertheless, according to the 2015 American College of Rheumatology (ACR) workforce survey, it was anticipated that women will make up to 57% of the United States rheumatology workforce by 2030 (2). This higher proportion of women in the ArLAR countries could be related to the unmet need for rheumatology specialists in these countries or the convenient lifestyle of rheumatologists.

As females are reported to treat 30% fewer patients than their male counterparts according to the 2015 ACR workforce survey or to stay at home in up to 30% in some Arab countries, the need for rheumatologists, in general, is expected to rise in the future if the female to male ratio increases (2). Thus, the concern of some of our interviewees about the future of rheumatology as a discipline is well-founded.

In general, the global number of rheumatologists per inhabitant (0.9/100,000) was similar to that in Latin America [1 per 106,838 inhabitants in a cross-sectional study from 19 countries in 2020 (15)] and most European countries [ranging from 0.5 to 0.93/100,000 (17)]. However, it was lower than the number of rheumatologists in the United States [1.9 per 100,000 inhabitants as per the 2015 American College of Rheumatology (ACR) Rheumatology Workforce Study Report (2)] and in France [3.8/100,000 (18)].

Despite the increasing women to men ratio in rheumatology, WR from the ArLAR region reported obstacles in academic advancement and leadership positions. At the international level, gender bias is obvious in the authorship of scientific articles. Overall, men have a higher publication rate than women across different scientific disciplines (3, 19). Moreover, women authors receive fewer citations (20, 21) and are also under-represented in first and senior authorship positions in articles published in medical journals, even in disciplines such as family medicine which are enriched for women practitioners (3, 22, 23). Nevertheless, according to this study, 49% of WR were involved in academic positions in the ArLAR countries, compared to 41% in the United States, according to a cross-sectional on 6,125 rheumatologists (6).

According to the interviews, it was perceived, first, that WR in the ArLAR countries tended to give a higher value to patient care and teaching and being close mentors to their students. This preference for being closer to the patients and to the students was also reported by others, as women seem to engage in clinician-educator tracks (1). Subsequently, it implied seeking fewer managerial and leadership positions. Second, priority given for family responsibilities in general, and motherhood duties in particular, was mentioned several times by the interviewees. In fact, 3 out of the 15 women mentioned that that their main achievement so far was raising a family. Also, having an optimal work-life balance was the primary obstacle faced by WR in the ArLAR countries, as it was mentioned by 6 out of the 15 interviewed women. This was not specific to the region but is reported by others, as demonstrated in the recent ACR 2021 session with WR leaders from all over the world (24).

As a response to these challenges, WR cited time management and early planning as a first solution. To help reach an optimal work-life balance, WR in the current study and the ACR session cited the role of family support and delegation as a second solution. In addition, good mentors were mentioned in most cases as having a positive impact on the career path of the WR, as were the hard work and pursuing higher levels of education, especially abroad when coming from developing countries. Finally, it was clear that there was a need to compromise and to set priorities, often citing family as a priority and sometimes advising to have personal time as well. As per most of the interviewees, the role of WR will continue to grow in the future. One of the means of growth may be their engagement on social media (SoMe). In a survey among 233 rheumatologists from 47 countries (25), 83% of respondents were active users on at least one SoMe platform, with an average weekly use of 6 hours and a majority of use related to work. Nevertheless, 72% of respondents were aged 30–39 years, and the results may not be extrapolated to older generations. Lack of knowledge about how to use SoMe was the most common reason for not using it, as found in studies conducted in Arab countries, where lack of technical knowledge of SoMe and how to use it positively and safely within privacy settings were the main barriers to using social networks (26). This highlights the need to understand better the value of SoMe and the opportunity to educate potential users on how to use it positively to facilitate learning and inter-professional relationships (27).

The current study has some limitations. The count of WR did not consider whether they worked part or full-time. Also, the level of academic positions was not available. Moreover, the selection criteria of the women for the interviews may be subjective as may be the estimation of what a “successful” WR is. Nevertheless, choosing women with leadership positions (dean of medicine, president of society, and head of department) could be used a surrogate for success definition. Also, since participants are already successful figures, the study may have missed the less successful WR who might have faced more difficult challenges not reported here.

## Conclusion

The proportion of WR varied widely across the ArLAR countries, with a mean of 56%, higher than the one reported from the rest of the world. Moreover, WR constituted 47% of the society’s board members. However, only 37% of the current society presidents were females, with an increasing trend in leadership positions over time. Roughly 49% of WR were involved in academia, but the level of involvement was not reported. Structured interviews indicate that WR place a high value on patient care and teaching, followed by research and publications. The primary reported gender-related challenge was balancing work with family demands. Moreover, some WR reported gender-related discrimination incurred by colleagues, patients, and administrations. Nevertheless, they provided valuable advice on overcoming these challenges and kept an optimistic view of their role in the future. As they embrace their growing role in the workforce, WR must benefit from all the tools provided to them, from learning from the experience of current women leaders in the field to using available technology such as social media platforms to empower them to reach gender equity.

## Data Availability Statement

The raw data supporting the conclusions of this article will be made available by the authors, without undue reservation.

## Ethics Statement

Ethical review and approval was not required for the study on human participants in accordance with the local legislation and institutional requirements. The patients/participants provided their written informed consent to participate in this study.

## Author Contributions

NZ, IH, and LE designed the study, collected the data, analyzed the results, drafted the manuscript, and revised it for intellectual content. All authors approved the version to be published and agreed to be accountable for all aspects of the work.

## Conflict of Interest

The authors declare that the research was conducted in the absence of any commercial or financial relationships that could be construed as a potential conflict of interest.

## Publisher’s Note

All claims expressed in this article are solely those of the authors and do not necessarily represent those of their affiliated organizations, or those of the publisher, the editors and the reviewers. Any product that may be evaluated in this article, or claim that may be made by its manufacturer, is not guaranteed or endorsed by the publisher.
