# Supplementary material for: Women in Rheumatology in the Arab League of Associations for Rheumatology Countries: A Rising Workforce
Source: Front Med (Lausanne). 2022 Jun 9;9:880285. doi: 10.3389/fmed.2022.880285 (PMC9218198; doi:10.3389/fmed.2022.880285)
Supplement: Supplementary file 1 [file Data_Sheet_1.PDF]

Supplementary data: Structured Questionnaire

What has brought you to rheumatology?

How did you become interested in rheumatology?

Did you have any mentors? If yes, how did you meet them? And who did they become your mentor?

What are the 3 top elements that shaped your career?

What 5 skills helped you to be a role model and inspiring doctor?

Did you face any obstacles because you're a woman? If yes, name the top 3.

How did you manage your work-life balance?

Do you have any tips for the new rheumatologists about that?

What has been your main achievement so far?

What is your current professional focus?

What is next for you? Is there a dream job as a rheumatologist?

How do you see the future of women rheumatologists in our region?

What is your MOTTO in life?
